# Impact of body mass index on procedural complications, procedure duration, and radiation dose in patients with atrial fibrillation undergoing radiofrequency ablation: A systematic review and meta‐analysis

**DOI:** 10.1002/clc.23398

**Published:** 2020-06-03

**Authors:** Menglu Liu, Kaibo Mei, Xiao Liu, Yujie Zhao

**Affiliations:** ^1^ Department of Cardiology The Seventh People's Hospital Zhengzhou Henan China; ^2^ Department of Anesthesiology The People's Hospital of Shangrao Jiangxi China; ^3^ Department of Cardiology The Second Affiliated Hospital of Nanchang University Jiangxi China

**Keywords:** atrial fibrillation, body mass index, complications, meta‐analysis, obesity, radiofrequency ablation

## Abstract

**Background:**

The association of body mass index (BMI) and procedure‐related factors in patients with atrial fibrillation (AF) after radiofrequency ablation (RFA) is still unclear.

**Hypothesis:**

BMI is associated with increased the radiation dose, procedure duration, and procedural complications.

**Methods:**

Prospective studies assessing BMI and procedure duration, radiation dose, and procedural complications in patients with AF after RFA were identified through electronic searches of PubMed, Embase, and the Cochrane Library database.

**Results:**

Ten studies with 14 735 participants undergoing RFA were included. Procedure duration was significantly longer in patients with overweight or obesity than in patients with normal BMI, with a mean difference (MD) of 0.95. Patients with overweight and obesity were exposed to a larger radiation dose, with standard MD of 1.71 and 1.98, respectively. There was no significant association between overweight or obesity and the risk of procedural complications (RR of 0.91 for overweight, 1.01 for obesity, 0.89 for stage I obesity, 1.00 for stage II obesity, and 0.94 for stage III obesity). Further analysis showed there was no significant difference regarding stroke or transient ischemic attack (overweight, RR: 0.92; obesity, RR: 1.02); cardiac tamponade (overweight, RR: 0.92; obesity, RR: 1.02); groin hematoma (overweight, RR: 0.62; obesity, RR: 0.40); or pulmonary vein stenosis (overweight, RR: 0.49; obesity, RR: 0.40) among BMI groups.

**Conclusion:**

Based on available evidence, we first showed that patients with overweight/obesity undergoing RFA experienced a significantly increased procedure duration and received a larger radiation dose than patients with normal BMI; however, there was no significant difference in procedural complications between patients with overweight/obesity and patients with normal BMI.

## 1 INTRODUCTION

Overweight/obesity is considered a risk factor for hypertension, stroke, coronary artery disease, and diabetes mellitus and poses a major challenge to the prevention of chronic diseases throughout the world.^1^, ^2^, ^3^ Atrial fibrillation (AF) is the most common sustained arrhythmia in clinical practice and is associated with an increased risk of stroke and all‐cause mortality. Obesity has been reported as an independent risk factor for new‐onset AF, being associated with a 20% higher risk of AF than normal weight.^4^ However, a number of clinical studies and meta‐analyses found that compared with patients with normal weight, patients with overweight or obesity did not have worse or even better outcomes among patients with AF, known as the “obesity paradox.”^5^, ^6^, ^7^ This phenomenon was also found in patients with obesity and other diseases; for example, in studies examining the rate of complications in patients undergoing percutaneous coronary intervention, the rate of complications was paradoxically found to decrease in patients with mild/moderate obesity.^8^, ^9^

In recent decades, radiofrequency ablation (RFA) of AF has emerged as an effective therapy and now has a Class I indication in symptomatic patients with drug‐refractory AF.^10^ However, the impact of obesity on procedural complications in patients undergoing RFA is inconsistent.^11^, ^12^, ^13^ Previous evidence demonstrated that the amount of radiation exposure for obese patients was more than twice that for patients with a normal BMI,^14^ which might result in a higher rate of complications for patients with obesity. Subsequently, a prospective study reported that the odds ratio of complications increased 3.1‐fold in those with morbid obesity.^12^ This association was confirmed by another observational cohort study of 3265 females.^11^ However, several cohort studies found no clear association.^11^, ^15^, ^16^ Therefore, whether overweight or obesity is associated with more complications than normal weight in patients with AF undergoing RFA is still under debate. From a practical standpoint, clarifying this point is of major importance for patients in decision making regarding whether to perform an ablation at the patient's current weight. Thus, in the current study, we sought to (a) assess the relationship between BMI and procedure duration and amount of radiation in patients undergoing RFA and (b) determine the association of BMI and complications in patients with AF after RFA. Differences in AF recurrence among different BMI groups were not within the scope of this study because all previous meta‐analyses have addressed this outcome.

## 2 METHODS

### 2.1 Literature search

We performed this meta‐analysis according to the PRISMA guidelines (Table S1 PRISMA checklist).^17^ Two authors (Yujie Zhao and Menglu Liu) independently searched the Cochrane Library, PubMed, and Embase databases for eligible studies until November 2019. Disagreements were resolved by consensus with a third investigator (Xiao Liu). Three groups of keywords (linked to BMI, AF, and catheter ablation) were combined using the Boolean operator “AND.” The first group of key words was linked to body mass (“body mass index” (BMI) OR “body weight” OR “obesity” OR “overweight” OR “central obesity”). The second group was linked to the type of diagnosis (“AF” OR “atrial flutter” OR “atrial tachycardia” OR “supraventricular tachycardia”). The third group of key words was linked to outcomes (“procedure time” OR “radiation” OR “complication” OR “pericardial effusion/tamponade” OR “stroke or transient ischemic attack” OR “groin hematoma” OR “pulmonary vein stenosis.” The last group of key words was linked to the intervention (“catheter ablation” OR “RFAs”). No language restrictions were applied to the literature search. The detailed search strategy is provided in Table S2 in Supplemental Material S1. In addition, this study has been registered with PROSPERO (International prospective register of systematic reviews)‐registration number‐CRD42019121373.

### 2.2 Outcome definitions and study selection

The primary endpoints were the procedure duration, the amount of radiation and procedure complications in patients with AF undergoing RFA. Secondary endpoints were major complications, including stroke or transient ischemic attack, pulmonary vein stenosis, cardiac tamponade, and groin hematoma. Studies were considered eligible if they (a) were designed as prospective epidemiological studies (cohort, nested case‐control, or clinical trial); (b) provided data on the assessment of at least one of the primary endpoints; and (c) for multiple publications/reports created from the same data, contained the longest follow‐up period or the largest number of cases. Certain publication types (eg, reviews, editorials, letters, conference abstracts, and animal studies) or studies with insufficient data were excluded from this study.

### 2.3 Data extraction and quality assessment

Two researchers independently assessed the eligibility of the literature according to the aforementioned inclusion criteria. All discrepancies were resolved through discussion or by a third researcher, as necessary. Two authors independently extracted the basic characteristics from each study, mainly including the first author, publication year, geographical location, study type, participants (sex, age, and sample size), duration of follow‐up, adjustments for confounders, categories of BMI and the number of cases and sample size for each BMI category.

The Newcastle‐Ottawa Scale (NOS) was used to evaluate the quality of all included studies.^18^ The validated NOS items with a total of nine stars involved three aspects, including the selection of cohorts, the comparability of cohorts, and the assessment of the outcome. In this meta‐analysis, an NOS score of ≥6 stars indicated a moderate‐ to high‐quality study; otherwise, the scores indicated a low‐quality study.^19^, ^20^

### 2.4 Statistical analyses

We expressed dichotomous outcome data as risk ratios (RRs) with 95% confidence intervals (CIs) and continuous outcome data as weighted mean differences (MDs) or standardized MDs with 95% CIs. BMI was a categorical variable according to the standard World Health Organization definition, in which “normal weight” was defined as a BMI of 18.5 to <25, underweight was defined as a BMI of <18.5, overweight was defined as a BMI of 25 to <30, obese was defined as a BMI of ≥30, grade 1 obesity was defined as a BMI of 30 to <35, grade 2 obesity was defined as a BMI of 35 to <40, and grade 3 obesity was defined as a BMI of ≥40. Count data for complications were used to generate unadjusted RRs and 95% CIs for different BMI groups. Given the heterogeneity in study designs and populations, the meta‐analysis was performed using a random‐effects model. To examine the influence of individual studies on the pooled results, a sensitivity analysis was performed by removing each study. To assess the heterogeneity across studies, the *I* ^2^ (95% CI) statistic was calculated with the following interpretation: low heterogeneity, defined as I^2^ < 50%; moderate heterogeneity, defined as *I* ^2^ = 50% to 75%; and high heterogeneity, defined as *I* ^2^ > 75%.^21^

Possible publication bias was assessed using Egger's test.^22^ All statistical analyses were performed using Review Manager (RevMan) software (Version 5.30, Nordic Cochrane Center, Rigshospitalet, Denmark) and Stata software (Version 14.0, Stata Corp LP, College Station, Texas). A *P*‐value <.05 was considered statistically significant.

## 3 RESULTS

### 3.1 Study selection

**FIGURE 1:**
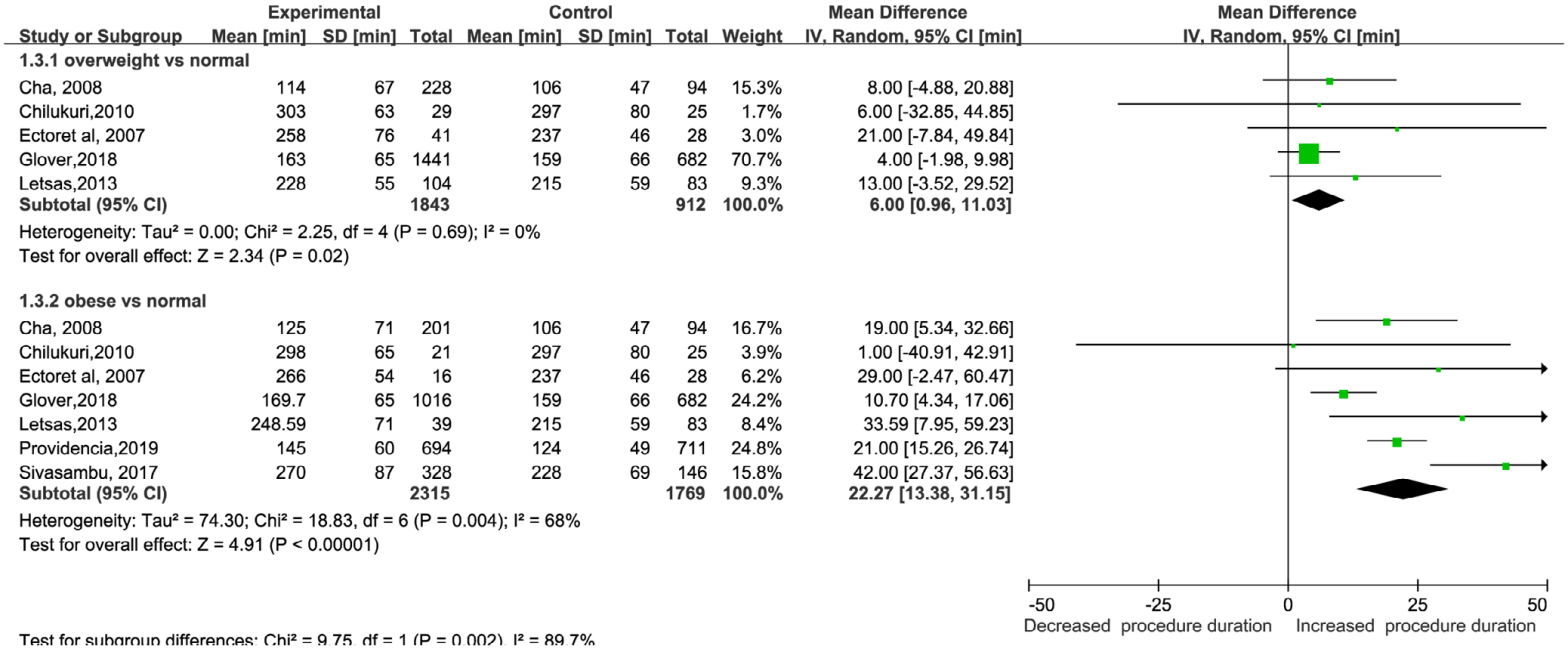
Forest plot of the association between body mass index and procedure duration in patients undergoing radiofrequency ablation. BMI: body mass index

As shown in Figure 1, we initially identified studies in the Cochrane Library (n = 13), PubMed (n = 173), and Embase (n = 321) databases (Figure S1). We excluded 201 studies based on screening the title or abstract, and the full text of the remaining studies was reviewed. After a quick screening of the full‐text articles, 20 were considered potentially eligible and were included for detailed evaluation, after which 10 were finally excluded for the following reasons: (a) outcomes of AF recurrence or quality of life (n = 3); (b) certain publication with no data (n = 5); and (c) reviews, comments or editorials (n = 2). Table S3 in Supplemental Material S1 provides the reasons for exclusion after the full‐text review. Finally, 10 studies^12^, ^13^, ^14^, ^15^, ^16^, ^23^, ^24^, ^25^, ^26^, ^27^ were included in this meta‐analysis.

### 3.2 Study characteristics and quality

Table 1 provides the detailed characteristics of the included studies. Overall, these studies were published between 2007 and 2019. The mean age ranged from 49 to 64 years. The sample sizes of the included studies varied from 85 to 3333, with a total of 14 290 individuals. Among the 10 articles, 7 were prospective cohort studies, and 3 were retrospective cohort studies. Seven were from North America (the United States and Canada), and three were from Europe. Eight studies reported procedural complications, seven studies reported the procedure duration, and four reported the amount of radiation.

**TABLE 1:** Basic characteristics of the 10 articles included in the meta‐analysis

Author, country	Source of participants	Study size	Mean age (y), male	Follow‐up (m)	Design	AF type		Outcomes reported	BMI data reported	Ablation strategy
						Paroxysmal%	Persistent%			
Cha et al,^23^ United States	Mayo Clinic Electrophysiology Laboratory	523	54, 84%	24	PC	58	42	Procedural complications Procedure duration Radiation dose	<25 25.0‐29.9 ≥30	SPVI
Ector et al,^14^ Belgium	University Hospital Gasthuisberg	85	49,75%	6 w	PC	NA	NA	Procedure duration Radiation dose	<25.0 25.0‐29.9 ≥30.0	CPVI
Chilukuri et al,^24^ United States	Johns Hopkins Hospital	109	60,78%	11	PC	67	33	Procedural complications Procedure duration	<25.0 25.0‐29.9 ≥30.0	SPVI
Jongnarangsin et al,^16^ United States	University of Michigan	324	57, 76%	12	PC	72	28	Procedural complications	<25 25‐29 ≥30	CPVI
Letsas et al,^25^ Europe	Evangelismos General Hospital of Athens	226	56, 81%	14.4 d	RC	64	36	Procedural complications Procedure duration Radiation dose	<25 25‐29.9 ≥30	CPVI
Winkle et al,^27^ United States	Sequoia Hospital	2715	64, 70%	12	RC	33	55	Procedural complications	<25 25‐30 30‐35 35‐40 ≥40	CPVI
Providencia,^26^ multicountry	Seven European Centers	2497	61, 72%	12	PC	58	33	Procedural complications Procedure duration	<25 25‐30 >30	
Glover et al,^15^ Canada	Cardiology, Queen's University	3333	58, 68%	20	PC	67	28	Procedural complications Procedure duration Radiation dose	<25 25‐30 >30	CPVI or SPVI
Shoemaker et al,^12^ United States	Vanderbilt University School of Medicine	512	61, 72%	NA	PC	NA	NA	Procedural complications	<40 ≥40	SPVI
Sivasambu et al,^13^ United States	Johns Hopkins Hospital	701	59, 72%	3	RC	59	41	Procedural complications Procedure duration	18.5‐25 25‐30 30‐40 >40	PVI

Abbreviations: AF, atrial fibrillation; CPVI, circumferential pulmonary vein isolation; NA, not available; PC, prospective cohort; PVI, pulmonary vein isolation; RC, retrospective cohort; SPVI, segmental pulmonary vein isolation.

The reporting quality of the included articles was high. All included studies obtained an NOS of ≥6 points (Table S4 in Supplemental Material S1).

### 3.3 Relationship between BMI and the duration of the procedure and amount of radiation

Six studies^13^, ^15^, ^23^, ^24^, ^25^, ^26^ that were included in this analysis reported the procedure duration. The procedure duration was significantly longer in patients with overweight or obesity, with an MD of 0.95 (95% CI: 1.69‐0.21) (Figure 1). There was no evidence of heterogeneity in the overweight (*I* ^2^ = 0, *P* = .69) groups and moderate heterogeneity in the obese (*I* ^2^ = 68%, *P* = .004) groups. However, the heterogeneity was not significant when Sivasambu et al was excluded (*I* ^2^ = 43%, *P* = .15), with a significant difference persisting between the obese and normal groups.

**FIGURE 2:**
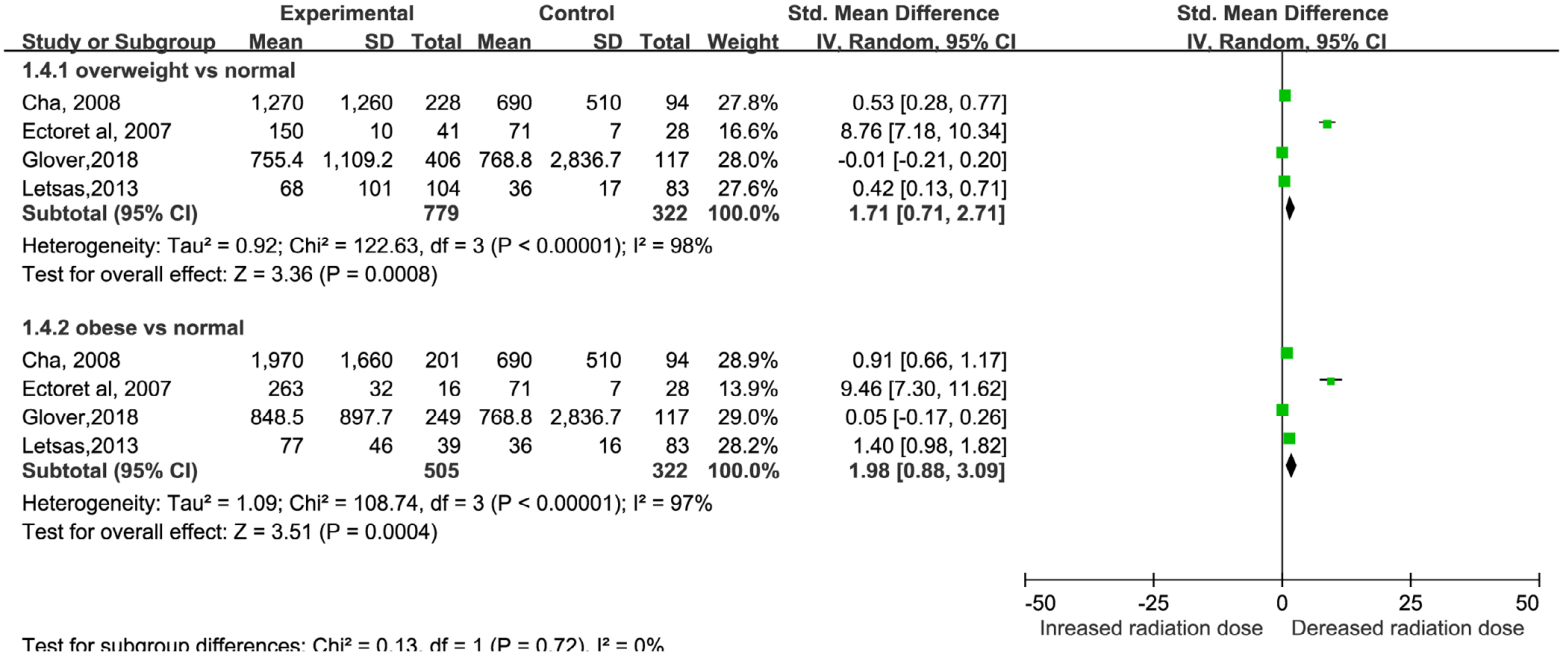
Forest plot of the association between body mass index and radiation dose in patients undergoing radiofrequency ablation. BMI: body mass index

Four studies^14^, ^15^, ^23^, ^25^ reported the radiation dose, with two studies using the unit of G and another two using the unit of uG × m^2^. Because a different unit was used across trials, the effect of the MD was used to summarize the results.^28^ We found that patients with overweight and obesity had a larger radiation dose than patients with normal BMI, with an SMD of 1.71 (95% CI: 0.71‐2.71) and 1.98 (95% CI: 0.88‐3.29), respectively (Figure 2). There was significant heterogeneity in the overnight (*I* ^2^ = 98%, *P* < .05) and obese (*I* ^2^ = 97%, *P* < .05) groups.

### 3.4 Relationship between BMI and the risk of procedural complications

**FIGURE 3:**
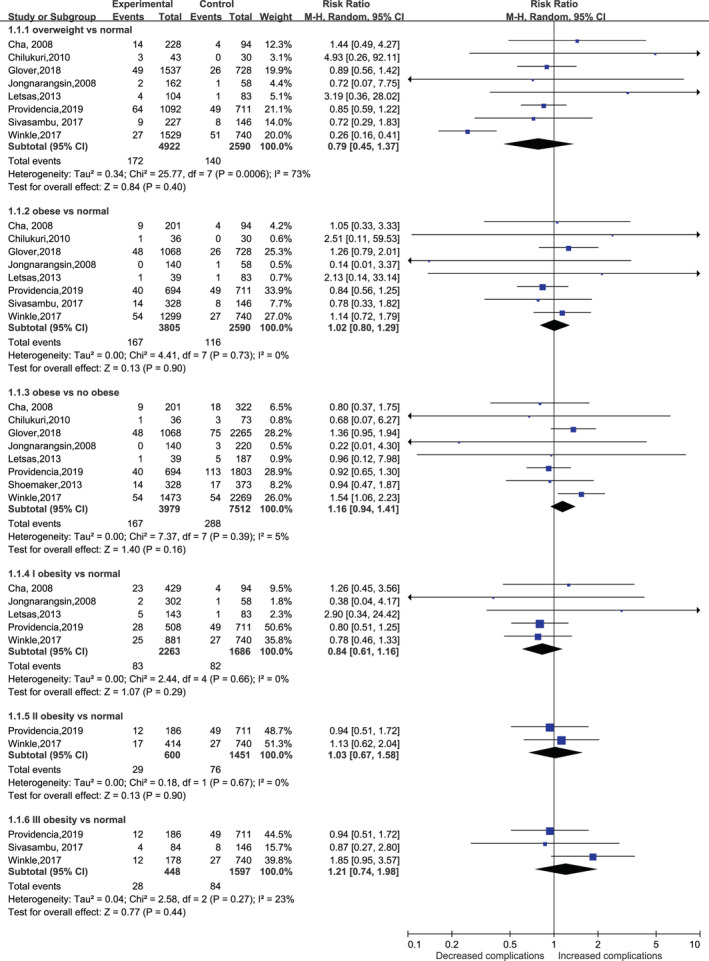
Forest plot of the association between body mass index and procedural complications in patients undergoing radiofrequency ablation. BMI: body mass index

Nine studies^12^, ^13^, ^15^, ^16^, ^23^, ^24^, ^25^, ^26^, ^27^ with 434 procedural complications, yielding an overall complication rate of 3.6% (434/11827), were included. Overall, neither overweight nor obesity increased the risk of procedural complications (RR of 0.91 for overweight, 1.01 for obesity, 0.89 for stage I obesity, 1.00 for stage II obesity, and 0.94 for stage III obesity), with no evidence of heterogeneity (*I* ^2^ = 0) (Figure 3). In addition, compared with the nonobese group, the obesity group also did not experience an increased rate of procedural complications(Figure 3).

Further analysis showed that there was no significant difference in the risk of stroke or transient ischemic attack (overweight, RR: 0.92, 95% CI: 0.40‐2.11‐1.88; obesity, RR: 1.02, 95% CI: 0.43‐2.46), cardiac tamponade (overweight, RR: 0.92, 95% CI: 0.40‐2.11‐1.88; obesity, RR: 1.02, 95% CI: 0.43‐2.46), groin hematoma (overweight, RR: 0.62, 95% CI: 0.27‐1.43; obesity, RR: 0.40, 95% CI: 0.10‐1.57), or pulmonary vein stenosis (overweight, RR: 0.49, 95% CI: 0.14‐1.66; obesity, RR: 0.40, 95% CI: 0.05‐3.11) in patients with overweight or obesity (Table 2 and Figures S2‐S5 in Supplemental Material S1).

**TABLE 2:** The association between BMI and the risk of major complications

BMI categories	Stroke or transient ischemic attack		Cardiac tamponade		Groin hematoma		Pulmonary vein stenosis	
	No. RRs	Summary RR (95% CI)	No. RRs	Summary RR (95% CI)	No. RRs	Summary RR (95% CI)	No. RRs	Summary RR (95% CI)
Overweight (25‐30)	6	0.92 (0.40, 2.11)	6	1.13 (0.55, 2.30)	3	0.62 (0.27, 1.43)	4	0.49 (0.14, 1.66)
Obese (≥30)	6	1.02 (0.43, 2.46)	6	1.28 (0.57, 3.89)	2	0.40 (0.10‐1.57)	4	0.40 (0.05, 3.11)

Abbreviations: BMI, body mass index; CI, confidence interval; RR, risk ratio.

### 3.5 Sensitivity analysis and publication bias

In the sensitivity analysis, the pooled results were not significantly changed when omitting one study at a time. Publication bias was not assessed because of the limited number of studies (N < 10), in accordance with the guidelines.^28^

## 4 DISCUSSION

In this comprehensive study, our study first showed that patients with overweight/obesity undergoing RFA experienced a significantly increased RFA procedure duration and radiation dose; however, there was no significant difference in total procedural complications between patients with overweight/obesity and patients with normal BMI. Specifically, further analysis showed that there was no significant difference in the risk of stroke or transient ischemic attack, cardiac tamponade, groin hematoma, or pulmonary vein stenosis among BMI groups.

However, our results cannot be explained by the fact that patients with overweight/obesity should undergo RFA regardless of their BMI. First, although overweight or obesity did not increase the risk of procedural complications, a significant increase in AF relapse was observed in many studies and meta‐analyses.^29^, ^30^ The increased risk of AF recurrence might result in elevated mortality, stroke, and AF burden. Second, long‐term outcomes (eg, all‐cause death, hospitalization) were not assessed in this study because of data restrictions. Notably, a recent study reported that although there was no difference in 3‐year mortality between different BMI groups, a slight difference was found in the cardiac hospitalization rate (*P* = .03) without adjustments for confounding factors.^31^ Therefore, further larger, well‐designed studies are needed to examine the relationship between BMI and long‐term outcomes in patients with AF undergoing RFA.

We previously reported that patients with high BMIs and AF did not have worse outcomes (eg, major bleeding, stroke) than patients with normal weight.^7^ In this study, we further investigated the impact of BMI on the risk of complications in patients undergoing RFA. Vascular complications were common in patients undergoing RFA, although several studies have shown that obesity increases anticoagulation,^32^ which suggest an increased risk of bleeding events (eg, cardiac tamponade). However, we found that the risk of several specific complications, including stroke or transient ischemic attack, cardiac tamponade, groin hematoma, and pulmonary vein stenosis, did increase in patients with overweight/obesity. Consistent with this, a large cohort study also found that BMI was not a predictor for any minor complications or major complications.^27^

It is known that morbid obesity significantly increases mortality in the general population.^33^ In addition, morbid obesity also significantly increased the rate of complications in patients undergoing PCI.^8^ Although we did not observe an increased rate of procedural complications in the morbid obesity groups in the current study, we cannot draw a conclusion with certainty based on the current evidence. First, the majority of previous works used a BMI cutoff of >30 kg/m^2^, which may have diluted a larger effect from the BMI >40 kg/m^2^ subset. A BMI cutoff >40 kg/m^2^ appeared to be the threshold at which the complication rate significantly increased, which has been reported by Shoemaker et al.^12^ Furthermore, because a few studies have specifically assessed morbid obesity in their analyses, the sample size for morbid obesity was relatively limited, and we cannot exclude the possibility that the effects of morbid obesity (BMI > 40) on the results would have been different if enough patients had been collected. Therefore, further larger, well‐designed studies are needed to clarify this issue.

### 4.1 Study limitations

Our meta‐analysis has some limitations that need to be mentioned. First, all of the included studies were observational. The inability to access individual patient data necessitated the use of a univariate meta‐analysis. A causal relationship between BMI and procedural complications could not be established due to confounding by other risk factors that may have influenced the results.^34^ For example, a previous study showed that in females, obesity increased the risk of procedural complications by 13%.^11^ Moreover, in another prospective cohort, Shoemaker et al^12^ found that female sex was a significant predictor of complications after adjusting for age and coronary artery disease. Second, there is significant heterogeneity between studies, which might be derived from the difference in baseline (eg, follow‐up duration, ablation strategy) characteristics of the patients. Third, we included only 10 studies that met our inclusion criteria. A small number of included studies may affect the reliability of the conclusions. Fourth, the experience of surgeon across the centers was varied and that is very important for the study outcomes, which also might influence our results.

## 5 CONCLUSION

Based on available evidence, we first showed that patients with overweight/obesity undergoing RFA experienced a significantly increased procedure duration and radiation dose; however, there was no significant difference in procedural complications between patients with overweight/obesity and patients with normal BMI. Further studies are required to determine the effect of morbidity obesity or weight reduction on the outcomes in patients with AF after RFA.

## CONFLICT OF INTEREST

The authors declare no potential conflict of interests.

## Supporting information


**Appendix** **S1.** Supporting Information.
