# Supplementary material for: Impact of body mass index on procedural complications, procedure duration, and radiation dose in patients with atrial fibrillation undergoing radiofrequency ablation: A systematic review and meta‐analysis
Source: Clin Cardiol. 2020 Jun 3;43(10):1067–75. doi: 10.1002/clc.23398 (PMC7534012; doi:10.1002/clc.23398)
Supplement: Supplementary file 1 — Appendix S1. Supporting Information. [file CLC-43-1067-s001.docx]

# Impact of body mass index on procedural complication, procedure duration, radiation dose in patients with AF undergoing catheter ablation: [A systematic review and meta-analysis.](https://www.ncbi.nlm.nih.gov/pubmed/31743918)

**SUPPLEMENTAL TABLES**

**Table S2: Search strategy
PubMed (search result=173)**

#1 (body mass index [Title/Abstract]) OR body weight [Title/Abstract] OR obesity [Title/Abstract] OR overweight [Title/Abstract] OR central obesity [Title/Abstract])

#2 (atrial fibrillation [Title/Abstract]) OR atrial tachycardia [Title/Abstract] OR supraventricular tachycardia [Title/Abstract])

#3 (catheter ablation [Title/Abstract]) OR radiofrequency ablations [Title/Abstract])

#4 #1 AND #2 AND #3

**Cochrane Library (search results=13)**

#1 (body mass index [Title/Abstract]) OR body weight [Title/Abstract] OR obesity [Title/Abstract] OR overweight [Title/Abstract] OR central obesity [Title/Abstract])

#2 (atrial fibrillation [Title/Abstract]) OR atrial tachycardia [Title/Abstract] OR supraventricular tachycardia [Title/Abstract])

#3 (catheter ablation [Title/Abstract]) OR radiofrequency ablations [Title/Abstract])

#4 #5 #1AND #2 AND #3

**EMBASE Database(search results=321)**

#1 body mass index:ab,ti AND ([article]/lim OR [article in press]/lim OR [conference abstract]/lim OR [conference paper]/lim)

#2 body weight:ab,ti AND ([article]/lim OR [article in press]/lim OR [conference abstract]/lim OR [conference paper]/lim)

#3 obesity:ab,ti AND ([article]/lim OR [article in press]/lim OR [conference abstract]/lim OR [conference paper]/lim)

#4 overweight:ab,ti AND ([article]/lim OR [article in press]/lim OR [conference abstract]/lim OR [conference paper]/lim)

#5 central obesity:ab,ti AND ([article]/lim OR [article in press]/lim OR [conference abstract]/lim OR [conference paper]/lim)

#6 #1 OR #2 OR #3 OR #4 OR #5

#7 atrial fibrillation:ab,ti AND ([article]/lim OR [article in press]/lim OR [conference abstract]/lim OR [conference paper]/lim)

#8 atrial tachycardia:ab,ti AND ([article]/lim OR [article in press]/lim OR [conference abstract]/lim OR [conference paper]/lim)

#9 supraventricular tachycardia:ab,ti AND ([article]/lim OR [article in press]/lim OR [conference abstract]/lim OR [conference paper]/lim)

#10 #7 OR #8 OR #9

#11 catheter AND ablation:ab,ti ([article]/lim OR [article in press]/lim OR [conference abstract]/lim OR [conference paper]/lim)

#12 radiofrequency AND ablations:ab,ti AND ([article]/lim OR [article in press]/lim OR [conference abstract]/lim OR [conference paper]/lim)

#13 #11 OR #12

#14 #6 AND #10 AND #13

**Table S3.** Studies excluded (n=10) with reasons

| **Studies excluded** | **Reasons** |
| --- | --- |
| Guijian, 2013(1) | Not the target methods: This is a meta-analysis |
| Hernandez, 2013(2) | Not the target methods: This is a meta-analysis |
| Kornej, 2013(3) | Without target data: This is an abstract without the RR and 95%CI |
| Prerfellner ,2004(4) | Not the target exposure and outcome: Quality of life and response to PVI |
| Wokhlu, 2010(5) | Not the target exposure and outcome: Long-Term Quality of life and recurrence AF after ablation |
| Zylla, 2016(6) | Not the target exposure: Sex-related Outcome and recurrence AF after ablation |
| Letsas,2013(7) | Without target data: did not provide the cases and sample size |
| Patel, 2010(8) | Without target data: did not provide the cases and sample size |
| Hoyt, 2011(9) | Not the target exposure: This was a case-control study and did not assess the impact of body mass index on complications after catheter ablation of atrial fibrillation |
| Mohanty,2011(10) | Not the target outcome: Long-Term Quality of life |

1. Guijian L, Jinchuan Y, Rongzeng D, Jun Q, Jun W, Wenqing Z. Impact of body mass index on atrial fibrillation recurrence: a meta-analysis of observational studies. Pacing Clin Electrophysiol. 2013;36(6):748-56. doi: 10.1111/pace.12106. PubMed PMID: 23437987.

2. Hernandez AV, Kaw R, Pasupuleti V, Bina P, Ioannidis JP, Bueno H, et al. Association between obesity and postoperative atrial fibrillation in patients undergoing cardiac operations: a systematic review and meta-analysis. Ann Thorac Surg. 2013;96(3):1104-16. doi: 10.1016/j.athoracsur.2013.04.029. PubMed PMID: 23932258.

3. Kornej J, Kosiuk J, Piorkowski C, Arya A, Sommer P, Rolf S, et al. Predictors for late recurrences of atrial fibrillation after catheter ablation in patients with early recurrences. European Heart Journal. 2013;34(suppl 1):P2324-P. doi: 10.1093/eurheartj/eht308.P2324.

4. Purerfellner H, Martinek M, Aichinger J, Nesser HJ, Kempen K, Janssen JP. Quality of life restored to normal in patients with atrial fibrillation after pulmonary vein ostial isolation. Am Heart J. 2004;148(2):318-25. doi: 10.1016/j.ahj.2004.03.036. PubMed PMID: 15309003.

5. Wokhlu A, Monahan KH, Hodge DO, Asirvatham SJ, Friedman PA, Munger TM, et al. Long-term quality of life after ablation of atrial fibrillation the impact of recurrence, symptom relief, and placebo effect. J Am Coll Cardiol. 2010;55(21):2308-16. doi: 10.1016/j.jacc.2010.01.040. PubMed PMID: 20488300.

6. Zylla MM, Brachmann J, Lewalter T, Hoffmann E, Kuck KH, Andresen D, et al. Sex-related outcome of atrial fibrillation ablation: Insights from the German Ablation Registry. Heart Rhythm. 2016;13(9):1837-44. doi: 10.1016/j.hrthm.2016.06.005. PubMed PMID: 27289011.

7. Letsas KP, Siklody CH, Korantzopoulos P, Weber R, Burkle G, Mihas CC, et al. The impact of body mass index on the efficacy and safety of catheter ablation of atrial fibrillation. Int J Cardiol. 2013;164(1):94-8. doi: 10.1016/j.ijcard.2011.06.092. PubMed PMID: 21726910.

8. Patel D, Mohanty P, Di Biase L, Sanchez JE, Shaheen MH, Burkhardt JD, et al. Outcomes and complications of catheter ablation for atrial fibrillation in females. Heart Rhythm. 2010;7(2):167-72. doi: 10.1016/j.hrthm.2009.10.025. PubMed PMID: 20022814.

9. Hoyt H, Bhonsale A, Chilukuri K, Alhumaid F, Needleman M, Edwards D, et al. Complications arising from catheter ablation of atrial fibrillation: temporal trends and predictors. Heart Rhythm. 2011;8(12):1869-74. doi: 10.1016/j.hrthm.2011.07.025. PubMed PMID: 21798230.

10. Mohanty S, Mohanty P, Di Biase L, Bai R, Dixon A, Burkhardt D, et al. Influence of body mass index on quality of life in atrial fibrillation patients undergoing catheter ablation. Heart Rhythm. 2011;8(12):1847-52. doi: 10.1016/j.hrthm.2011.07.005. PubMed PMID: 21740879.

**Table S4**. Quality assessment of included studies

| Author  (Publication Year) | Newcastle-Ottawa Scale | | | | | | | | | |
| --- | --- | --- | --- | --- | --- | --- | --- | --- | --- | --- |
|  | Selection | | | Comparability | | | Outcome | | | Total |
|  | a | b | c | d | e | f | g | h | i |  |
| Cai et al,2011 | 1 | 1 | 1 | 1 | 0 | 0 | 1 | 1 | 1 | 7 |
| Chilukuri et al, 2010 | 1 | 1 | 1 | 1 | 0 | 0 | 1 | 1 | 1 | 7 |
| Jongnarangsin et al,2008 | 1 | 1 | 1 | 1 | 0 | 0 | 1 | 1 | 1 | 7 |
| Letsas et al,2008 | 1 | 1 | 1 | 1 | 0 | 0 | 1 | 1 | 1 | 7 |
| Glover et al,2018 | 1 | 1 | 1 | 1 | 0 | 0 | 1 | 1 | 1 | 7 |
| Winkle et al, 2017 | 1 | 1 | 1 | 1 | 0 | 0 | 1 | 0 | 1 | 6 |
| Ector et al, 2007 | 1 | 1 | 1 | 1 | 1 | 1 | 1 | 0 | 1 | 8 |
| Provid^encia et al, 2019 | 1 | 1 | 1 | 1 | 0 | 0 | 1 | 1 | 1 | 7 |
| Shoemaker et al ,2013 | 1 | 1 | 1 | 1 | 0 | 0 | 1 | 1 | 1 | 7 |
| Sivasambu et al,2017 | 1 | 1 | 1 | 1 | 0 | 0 | 1 | 0 | 1 | 6 |

1. Representativeness of the exposed cohort.
2. Selection of the non-exposed cohort.
3. Ascertainment of exposure.
4. Demonstration that outcome of interest was not present at start of study.
5. Comparability of cohorts on the basis of the design or analysis (adjusted for age).
6. Comparability of cohorts on the basis of the design or analysis (adjusted for any other factor).
7. Assessment of outcome.
8. Was follow-up long enough for outcomes to occur. (>30 days).
9. Adequacy of follow-up of cohorts.


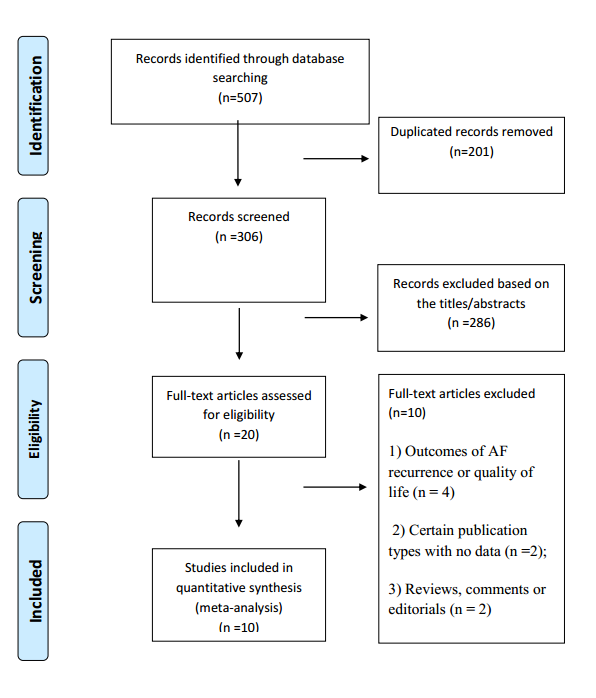


**Figure S1. Overview of the research strategy.**


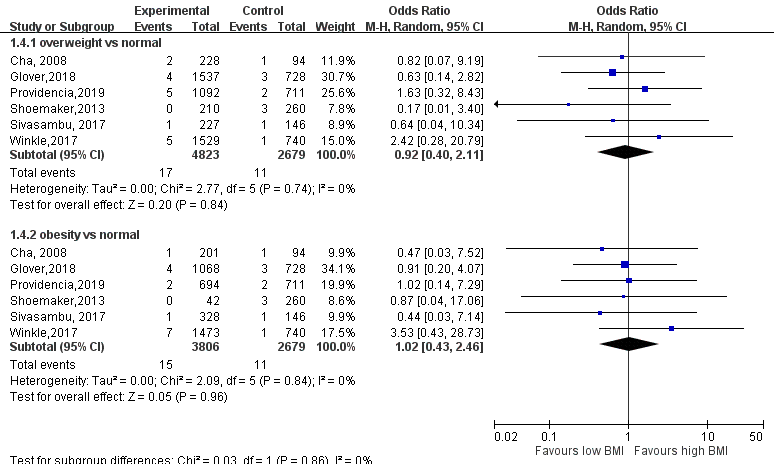


**Figure S2: Forest plot of the association between BM and stroke or transient ischemic attack in patients with AF undergoing radiofrequency ablation**


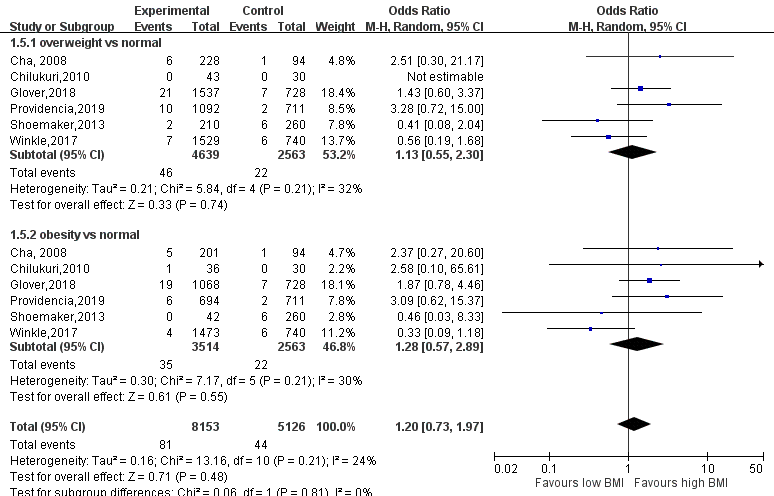


**Figure S3: Forest plot of the association between BM and cardiac tamponade in patients with AF undergoing radiofrequency ablation**

**
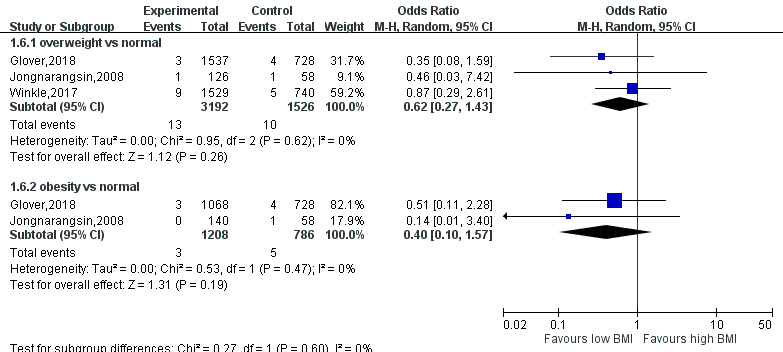
**

**Figure S4: Forest plot of the association between BM and groin hematoma in patients with AF undergoing radiofrequency ablation**


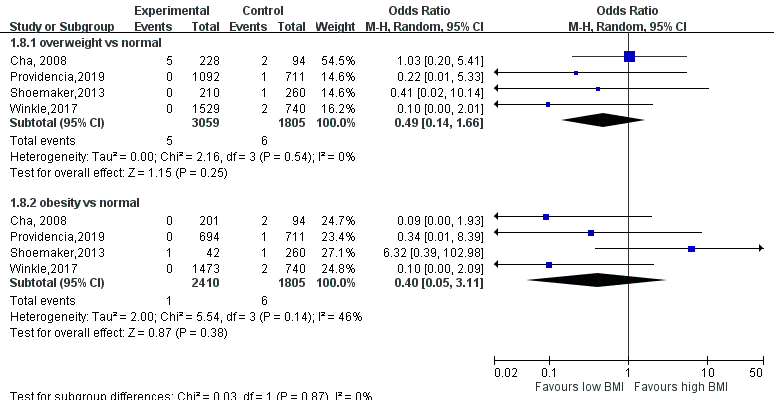


**Figure S5: Forest plot of the association between BM and pulmonary vein stenosis in patients with AF undergoing radiofrequency ablation**
